# The Structure of Negative Social Ties in Rural Village Networks

**DOI:** 10.15195/v6.a8

**Published:** 2019-03-06

**Authors:** Alexander Isakov, James H. Fowler, Edoardo M. Airoldi, Nicholas A. Christakis

**Affiliations:** a Alexander Isakov Yale Institute for Network Science, Yale University; Department of Sociology, Yale University. E-mail: alexander.isakov.11@gmail.com; b James H. Fowler Department of Medicine, University of California, Sand Diego; Political Science Department, University of California, San Diego. E-mail: fowler@ucsd.edu; c Edoardo M. Airoldi Department of Statistical Science, Fox School of Business, Temple University; Department of Statistics and Institute for Quantitative Social Sciences, Harvard University. E-mail: airoldi@fas.harvard.edu; d Nicholas A. Christakis Department of Ecology and Evolutionary Biology, Yale University; Department of Statistics and Data Science, Yale University. E-mail: nicholas.christakis@yale.edu

**Keywords:** negative ties, antagonistic ties, animosity, enemies, social networks, network structure

## Abstract

Negative (antagonistic) connections have been of longstanding theoretical importance for social structure. In a population of 24,696 adults interacting face to face within 176 isolated villages in western Honduras, we measured all connections that were present, amounting to 105,175 positive and 16,448 negative ties. Here, we show that negative and positive ties exhibit many of the same structural characteristics. We then develop a complete taxonomy of all 138 possible triads of two-type relationships. Consistent with balance theory, we find that antagonists of friends and friends of antagonists tend to be antagonists; but, in an important empirical refutation of balance theory, we find that antagonists of antagonists also tend to be antagonists, not friends. Finally, villages with comparable levels of animosity tend to be geographically proximate. Similar processes, involving social contact, give rise to both positive and negative social ties in rural villages, and negative ties play an important role in social structure.

ANIMOSITY in social relations may provide a sort of repulsive force, pushing other people in groups together. And so negative ties may affect the surrounding network structure based on friendship. A basic theoretical conceptualization of this was codified by Georg Simmel a century ago (Simmel 1950) and advanced by Heider (1946) and Cartwright and Harary (1956). In considering very small groups composed of just three people, they proposed that certain kinds of triads can be seen as balanced or stable and others as unbalanced or unstable (Figure 1). Simply stated, friends should have the same friends and also the same “enemies.” Your friend’s antagonist cannot be your friend in a stable triad (Davis 1963).

**Figure 1:**
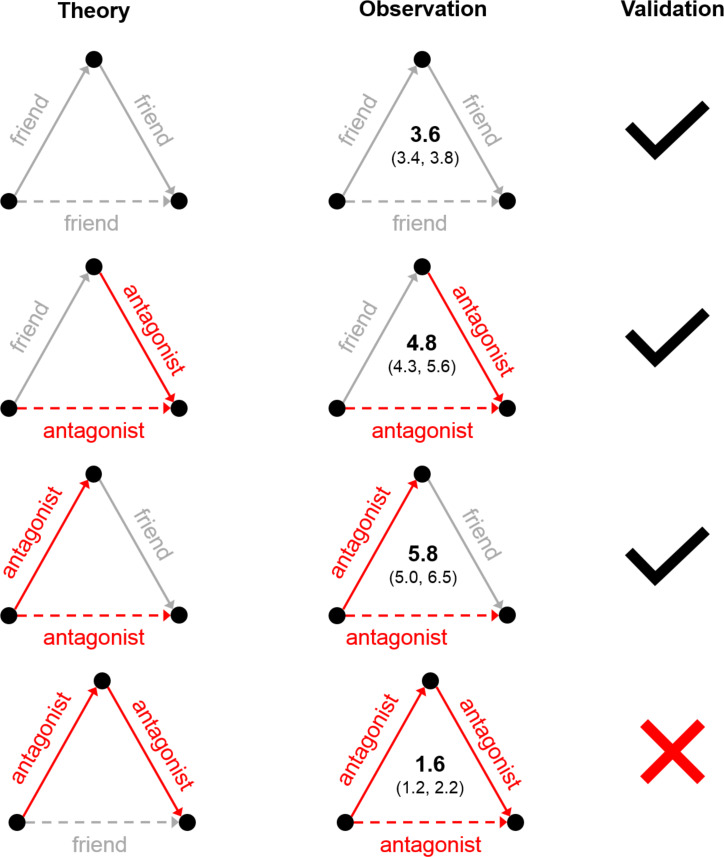
Experimental observations of balance theory. The first column shows the expected balanced triads from traditional balance theory. In the top triangle, we expect the friend of your friend to be your friend (dashed gray line). In the next two triangles, we expect the antagonist of your friend and the friend of your antagonist to be your antagonist (dashed red line). Finally, balance theory predicts that the antagonist of your antagonist is expected to be your friend (dashed gray line) rather than your antagonist. The second column shows results in a large data set of 105,175 positive and 16,448 negative ties. The inset numbers indicate the relative probability of the observed tie compared to chance. The smaller numbers represent the 99.5 percent confidence interval. Column 3 shows that we validate the first three rules of balance theory but that the fourth rule is not empirically confirmed. Rather, in reality, “the enemy of my enemy is my enemy.”

In graph-theoretic terms, this translates to requiring the product of the signs in a triangle to be positive; triangles that violate this property are deemed unbalanced. “Balance theory” thus postulates that agents seek to balance the valence in their local social systems. This can happen either by a change in sentiment, such as coming to see a friend as an antagonist, or by a change in structure, such as forming or cutting a tie (Rawlings and Friedkin 2017). Over time, it is theorized, one’s friends’ friends will tend to become one’s friends, one’s antagonists’ friends will tend to become one’s antagonists, one’s friends’ antagonists will tend to become one’s antagonists, and one’s antagonists’ antagonists will tend to become one’s friends (Rapoport 1963; Davis 1967). Friendship and animosity are thus intertwined in human social networks, and such adages are found in many cultures (Rawlings and Friedkin 2017). But balance theory also has implications for larger networks that surpass just three people (when indeed such networks afford both positive and negative interactions), and fundamental empirical questions arise regarding the geodesic location and role of negative ties within social networks in ways that move beyond triads. Recent work on the structure of negative social ties has expanded this context to workplaces, classrooms, the Internet, and the natural world.

Antagonism in humans is an old and deeply studied topic. Classic ethnographies have highlighted the importance of antagonistic interactions between groups and between pairs of individuals, including as an explanation for homicide (Chagnon 1988). But the actual sociocentric mapping of negative ties in parallel with positive ties in social networks is uncommon (Everett and Borgatti 2014; Offer and Fischer 2017), especially compared to the many thousands of empirical articles examining social networks based on positive ties. One study examined negative ties in 129 people in a sample of 31 urban communes in the United States from the 1970s (Rawlings and Friedkin 2017), and a classic study by Sampson (1969) on 18 novitiate monks collected information about members of the group who were disliked. Studies have also mapped helpful and adversarial relationships in classrooms (Mouttapa et al. 2004; Huitsing and Veenstra 2012; Huitsing et al. 2012) and workplaces (Labianca and Brass 2006; Xia, Yuan, and Gay 2009; Gerbasi et al. 2015), typically involving samples from a dozen to a few hundred people. In the context of the workplace, negative interactions can affect performance (de Jong, Cur¸seu, and Leenders 2014), and not always adversely (Marineau, Labianca, and Kane 2016). And negative ties may be a particular problem in social situations that people cannot easily escape, such as workplaces or rural villages.

Other recent work has examined negative ties in the networks formed by wild mammals (Lea et al. 2010; Ilany et al. 2013) or online interactions (Facchetti, Iacono, and Altafini 2011). One important study of 18,819 players in a massive online game involving artificial interactions found that positive ties (such as sending a private message) substantially outnumbered negative ones (such as placing a targeted bounty) and that positive ties were reciprocated much more often than negative ties (Szell, Lambiotte, and Thurner 2010). Studies of websites focused on social interactions around product reviews (Massa and Avesani 2005; Guha et al. 2014) or controversial opinions (Brzozowski, Hogg, and Szabo 2008) found that explicitly adding the ability to “distrust” (dislike) content offered by others enhanced the performance of prediction algorithms. In some circumstances, online users can even designate other users as “friends” or “foes,” and one study of this practice provided support for multiplicative transitivity (“the enemy of my enemy is my friend”; Kunegis, Lommatzsch, and Bauckhage 2009). More generally, there has been increasing interest in understanding the structure of negative ties both empirically and algorithmically through mining signed networks from social media (for an overview, see Tang et al. 2016). Of course, the online environment is different from face-to-face communities, as there is no practical limit on nominating enemies or friends, and the underlying contexts of empirical studies are typically highly specific.

Models of network formation, which often build on assumptions about myopic agents interacting, also only rarely include negative interactions (Antal, Krapivsky, and Redner 2005; Kossinets and Watts 2006; Ludwig and Abell 2007; Christakis et al. 2010; Marvel et al. 2011). In such models, global network structure at any point in time is seen as emerging from the dynamic local decision rules of individual agents. For instance, if agents tend to attach to more popular actors, scaling can emerge in the degree distribution of the graph (Barabasi and Albert 1999); if people generally form connections with people who are similar, homophily is apparent (McPherson, Smith-Lovin, and Cook 2001), or if people choose to form ties with friends’ friends, they close sets of previously open triads (Davis 1963; Louch 2000). Some of these properties, such as triadic closure and degree assortativity (the tendency of people with similar numbers of connections to be connected), have been theorized to play a role in human evolution, as they may affect the ability of groups to coordinate activities or resist epidemics (Fowler, Dawes, and Christakis 2009). Recently, researchers have used such structural insights to motivate new algorithms for predicting negative ties and generating signed networks (Derr, Aggarwal, and Tang 2018; Wang et al. 2018).

But much less is theorized or known about the fundamental properties of antagonistic networks. For instance, one might think that pairs of individuals who report negative interactions should be dissimilar (not similar) to each other or that people with many friends should have few antagonists. Also, in principle, negative ties also need not share the same structural properties of positive ties. This is especially likely to be the case if antagonistic ties served a different overall purpose than friendship ties over the course of human evolution (Hruschka 2010; Apicella et al. 2012; Christakis and Fowler 2014). Still, the extent and geodesic location of negative ties is important to human groups because negative ties may affect the structure of social networks, possibly affecting the ways people circulate information, maintain cohesion, or produce and distribute resources.

Rural villages—especially if they are isolated—provide an appealing laboratory in which to study the prevalence, properties, and role of negative ties in social networks. These are face-to-face communities, and villagers typically reside for long periods with others in one place, can come to know everyone personally, and cannot escape unpleasant interactions. Social-network structure may also be especially relevant to collective action in such small-scale, demanding settings, such as the ability of villagers to adopt new practices related to public health or to maintain resources held in common (such as a health outpost).

## Results

Here, we performed a large-scale, comprehensive, sociocentric network study of 24,696 people aged 12 to 93 years in 176 geographically isolated villages in western Honduras (see online supplement Table S1 for summary statistics; see Shakya et al. [2017] for additional details). We measured both positive (friendly) and negative (antagonistic) ties among all the residents within each village, also allowing subjects to offer no opinion of each other (and hence to be “strangers”). By design, all the networks were solely within-village networks (see the Methods section). The villages varied in size from 42 to 512 participants (mean = 140.3; SD = 85.6).

**Figure 2:**
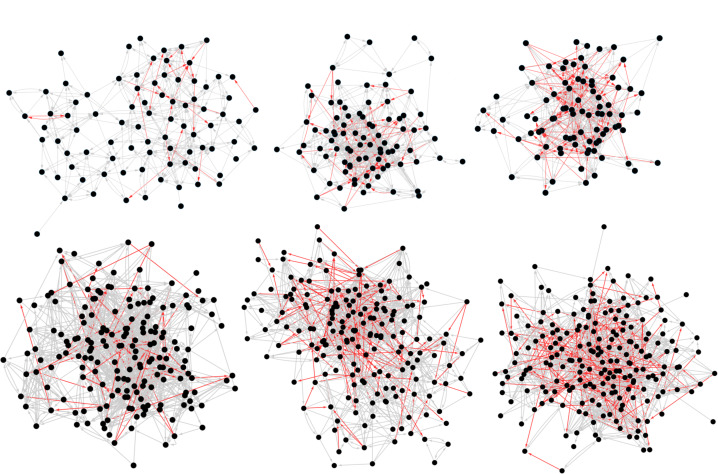
Six village networks of varying size and levels of antagonism. Friend ties are gray, and antagonistic ties are red. The top row represents three smaller villages (N = 86, 87, and 87 from left to right), and the bottom row represents three larger villages (N = 204, 188, and 184) chosen from the 176 villages. The left column shows villages with low animosity (8.5–9.6 percent), the middle column shows villages with medium animosity (17.2–21.6 percent), and the right column shows villages with high animosity (40.0–32.2 percent) as measured by the ratio of negative ties to positive ties in the villages.

We ascertained 105,175 positive ties and 16,448 negative ties; hence, the overall prevalence of negative ties was 15.6 percent (measured as the number of negative ties to all ties). Figure 2 shows whole network graphs for 6 sample villages, including both positive and negative ties. On average, people nominated 4.3 friends (SD = 2.6), with a range of 0 to 29; 86.7 percent had between one and seven friends, and 2.4 percent reported having no friends. On the other hand, on average, people identified just 0.7 other people they did not like (SD = 1.2), and a total of 65.4 percent of the subjects reported having no antagonistic ties. Although the majority of people reported not disliking anyone, 31.9 percent of these people actually were disliked by others.

**Figure 3:**
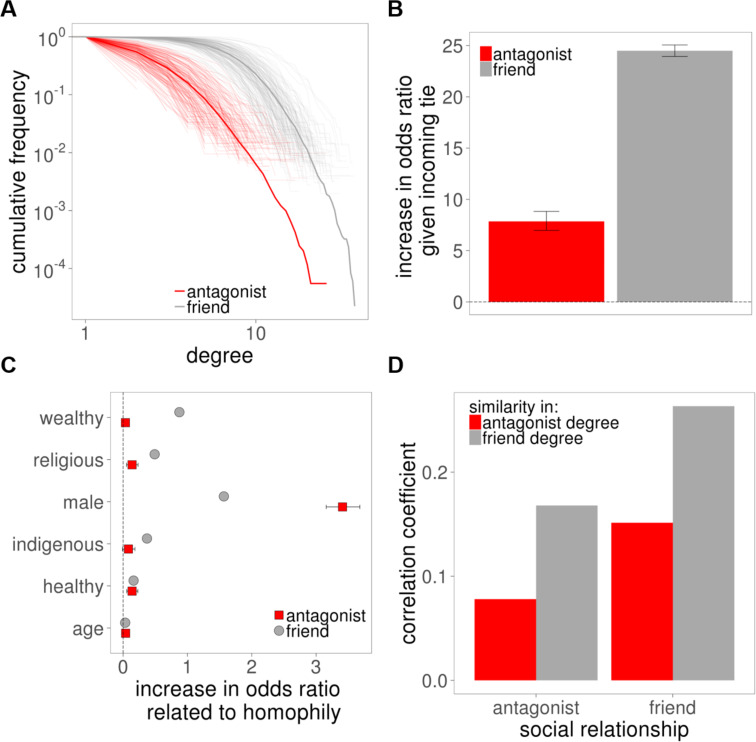
Properties of positive and negative ties. (A) Degree distribution of friend and antagonistic ties across 176 villages with 105,175 positive and 16,448 negative ties. Transparent lines represent observed cumulative degree distributions for friends (gray) and antagonists (red) for each symmetrized village network. Solid lines represent the average distribution. Axes are log scale. (B) Increase in the odds of the ego naming the alter as an antagonist (friend) given that the alter named the ego as an antagonist (friend) based on estimates from a dyadic model with fixed effects (see the Methods section). (C) Increase in the odds of an outgoing tie (*x* axis) based on dyadic models of social ties indicate how much a one-unit change in homophily (similarity) in each of the variables shown (*y* axis) is associated with increased odds of a social tie. Gray circles indicate friends, and red squares indicate antagonists. Black lines around estimates indicate 99.5 percent confidence intervals (see the online supplement). Both friends and antagonists show homophily on the indicated traits. (D) Pearson correlations with respect to the network degree of connected individuals. Red bars correspond to similarity in antagonist degree, and gray bars correspond to similarity in friend degree for the given social relationship (*x* axis) between pairs of nodes. Among pairs of friends, the number of friends and antagonists is correlated, as it is among pairs of antagonists.

Interestingly, negative ties share many important structural characteristics with positive ties. Degree distributions for both positive and negative ties across all villages are skewed, and they have a similar shape (Figure 3A). The primary difference is a leftward shift in the antagonistic distributions, reflecting the substantially lower overall incidence of negative ties. We also found that the antagonistic tie distributions in the villages exhibit greater variance than friendship ties, using a procedure that conditions on the mean in a simple linear regression or using an alternative procedure involving a simulation in which we randomly deleted friendship ties until they matched the number of negative ties (see Figure S1 and Table S14 in the online supplement). This suggests that the environmental, social, or biological forces affecting the formation of positive ties may generate greater conformity than those affecting negative ties.

Both friendly and antagonistic ties exhibit reciprocity, with an observed probability of reciprocation of 33.6 percent and 5.1 percent, respectively. In model estimates (see the online supplement), the odds of an individual naming a person as a friend if they were also named as a friend by that person increased by 24.5 times (99.5 percent confidence interval [CI]: 23.9, 25.1), and the odds increased by 7.9 times (99.5 percent CI: 7.0, 8.8) for a person naming another as an antagonist if they were also named as an antagonist by that person (Figure 3B; see online supplement Table S2). In other words, people’s odds of reciprocating friendship were three times higher than their odds of reciprocating antagonism; this may relate to the fact that people typically tell each other when they like each other but not when they dislike each other.

Negative ties also exhibit homophily (Figure 3C). People tend to dislike people who are similar, not dissimilar. For example, using logistic regression models (see the online supplement), individuals’ odds of being antagonists with someone of the same sex increased 3.4 times (99.5 percent CI: 3.2, 3.7), of being antagonists with someone of the same health status increased 0.1 times (99.5 percent CI: 0.0, 0.2), and of being antagonists with someone for each year of similarity in age increased 0.04 times (99.5 percent CI: 0.04, 0.04) (see online supplement Tables S3–S8). Thus, the processes that bring people of similar traits into greater contact may generate not just friendship ties but antagonistic ties as well (Fu et al. 2012).

Positive ties and negative ties are also similar with respect to degree assortativity. People with more friends tend to be friends with one another (Pearson correlation *ρ* = 0.263) but also antagonistic with one another, too (*ρ* = 0.168). Antagonistic ties also exhibit assortativity within the negative-tie network itself; people with many negative ties were preferentially likely to be antagonists with each other (*ρ* = 0.078), but also, people with more negative ties tend to be friends with each another (Pearson correlation *ρ* = 0.151; Figure 3D).

The similarity in the foregoing aspects of positive and negative ties may relate to an underlying propensity to social activity. A person who never interacts with others will have neither positive nor negative ties, so the tendency to be socially active might drive the formation of both types of ties. We find evidence for this in regression models of tie presence (see online supplement Tables S9 and S10). Each additional friendship nomination a person gives increases the odds that they will also name an antagonist by 0.1 times (99.5 percent CI: 0.1, 0.12).

In spite of the similarity between positive and negative ties, graphs of such ties differ in one important respect: transitivity. As predicted by balance theory, we find that positive ties are much more transitive than negative ties. Across all observations, the transitivity in friends is 0.228, consistent with past work (Apicella et al. 2012), but transitivity in enemies is 0.038. Due solely to chance, these two numbers would be 0.074 (99.5 percent CI: 0.072, 0.075) and 0.016 (99.5 percent CI: 0.012, 0.020), respectively (see the Methods section).

We find a similar pattern of results in the regression models of tie presence (online supplement Table S11). Each additional friend that two people have in common increases the odds that they will be friends with one another by 2.3 times (99.5 percent CI: 2.2, 2.3). But we find that this also increases the odds that they will be antagonistic by 0.2 times (99.5 percent CI: 0.2, 0.3). Likewise, each additional antagonist two people have in common increases the odds they will be friends with one another by 0.9 times (99.5 percent CI: 0.8, 1.0), and it also increases the odds that they will be antagonists by 0.7 times (99.5 percent CI: 0.5, 0.9). These results, like the others above, would appear to relate to the necessity of knowing someone in order to come to dislike them.

The large size of our data set allowed us to perform a taxonomic survey of all possible triadic combinations of face-to-face relationships, including rare ones that would not easily be observed in smaller samples. We categorized an exhaustive triad census in which we characterized whether each of the six possible directed ties among three nodes existed (allowing for positive, negative, and no ties [i.e., friend, antagonist, and stranger]; see the online supplement). We then complement this survey with a probabilistic assessment of how surprising each triad is with respect to a baseline network model for positive and negative ties, which we calibrated on the observed networks.

**Figure 4:**
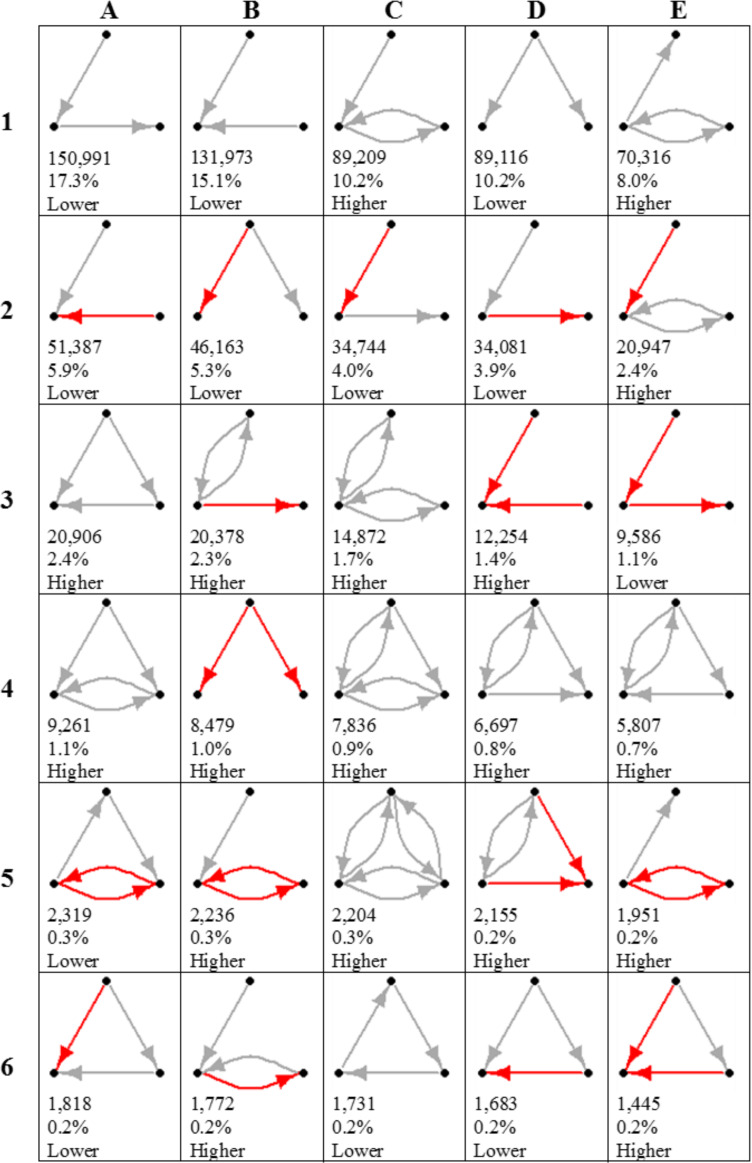
Illustration of the top 30 empirically observed connected triads. This shows the 30 most numerous of the 132 possible directed connected triads (those without a completely disconnected node) across all villages ordered by the total number of observations. Gray ties represent friendship; red ties represent antagonism. Total counts are shown below each triad as well as their percentage representation among connected triads. Comparison to simulated random networks (N = 1,000) is shown at the bottom of each cell; “higher” means that the triad appears in villages more often, and “lower” means that the triad appears in villages less often than is due to chance alone (*p <* 0.005). Letters A through E (top row) and numbers 1 through 6 (left column) are guides for easier cell identification. The full Heterogenous Triad Census is shown in the online supplement (Table S23).

There are 138 possible structural triads in such a census from these six types of ties (online supplement Table S23), of which 132 are connected triads (those without a disconnected node). This may be contrasted to the long-studied subset of just 16 permissible triads (13 of which are connected triads) seen with solely directed friendship ties (Davis 1963; Davis and Leinhardt 1972). The top 30 most commonly observed connected triads in our data are shown in Figure 4. The top 20 connected triads account for more than 95 percent of connected triads (the top 30 account for 97.8 percent), suggesting that a relatively small number of triads dominate social interaction. Of these, unbalanced triads (e.g., A5 and A6, wherein the friend of a friend is an antagonist, or D6, wherein the antagonist of a friend is a friend) appear significantly less than by chance (*p <* 0.005), whereas balanced triads (e.g., C4 and C5, wherein the friend of a friend is a friend, or E6, wherein a friend’s antagonist is an antagonist) appear more frequently.

We carried out a simulation study to compare empirically observed values to values generated in 1,000 randomly generated “worlds” of 176 villages each, where the total number of positive and negative ties (and people) were kept constant within each village. The results reveal that the ratio of friends to antagonists among friends’ friends in our data is about 3.6 times (99.5 percent CI: 3.4, 3.8) what we would expect due to chance (Figure 1). This confirms positive balance. In addition, the ratio of antagonists to friends was 4.8 times chance (99.5 percent CI: 4.3, 5.6) for friends’ antagonists, and the ratio of antagonists to friends was 5.8 times chance (99.5 percent CI: 5.0, 6.5) for antagonists’ friends. These results confirm negative balance. But we find an important exception to balance theory (comporting with an early proposal by Davis [1967] regarding weak structural balance): Antagonists’ antagonists are about 1.6 times *more* likely than chance to be antagonists rather than friends.

Although balance is observed for both positive and negative ties, we find a stronger effect for friends. Subjects are 1.42 times more likely to form any kind of relationship (positive or negative) with their friends’ social contacts than with their antagonists’ social contacts, and this value lies outside the 99.5 percent confidence interval of the randomly permuted networks (mean = 0.98; 99.5 percent CI: 0.93, 1.02). These results suggest that there are two mechanisms promoting positive balance in human social networks: (1) an attentional mechanism, whereby subjects pay more attention to their friends’ social contacts; and (2) an affective mechanism, whereby subjects are more likely to like their friends’ friends than to dislike their friends’ antagonists.

**Figure 5:**
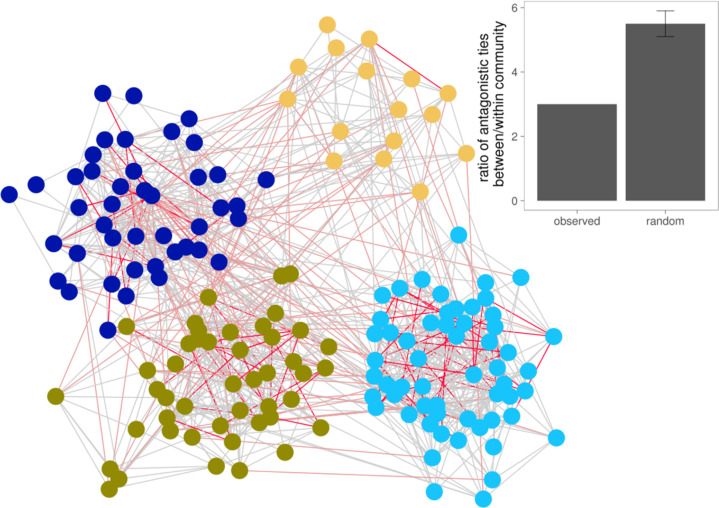
Illustration of between-community versus within-community ties. In this network from one village (N = 162 people), node colors represent distinct communities detected by a fast-greedy algorithm, gray ties represent friendship, light-red ties represent between-community antagonistic ties, and dark-red ties represent within-community antagonistic ties. In the observed data, there are three times more antagonistic ties between communities than within communities. But this is less than we would expect due to chance, suggesting that frequent social contact within communities may contribute to the formation of antagonistic ties. The inset shows the real mean ratio (left bar) compared to the mean and 99.5 percent confidence interval of more than 1,000 sets of 176 random networks of villages (right bar; see the Methods section).

Given the evidence for the existence of positive and negative balance in these village networks, we evaluated the structural location of negative ties not only in triadic relationships but also in higher-order network features, such as network communities within each village. We used a fast-greedy community-detection algorithm to partition each village network based on their positive ties alone. We then studied the location of antagonistic ties relative to these communities (for an example, see Figure 5). Negative ties are about 3.0 times more likely to lie between communities than within communities, suggesting that antagonistic connections may contribute to fissures within networks (or vice versa). However, that ratio is *less* than we would expect due to chance. In 1,000 randomly permuted networks, antagonists were 5.5 times (99.5 percent CI: 5.1, 5.9) more likely to exist between communities than within them. In other words, although the great majority of antagonistic ties are between groups, the probability that a given tie within a group is negative is higher than the probability between groups. This result is consistent with the finding that antagonistic ties exhibit homophily and depend on social interactions, and it gives still more credence to the adage “familiarity breeds contempt.” A sensitivity analysis using additional community-detection methods (see the online supplement) confirms that our substantive results are not sensitive to the method used (Clauset, Newman, and Moore 2004; Newman and Girvan 2004; Blondel et al. 2008).

Next, we studied whether two individuals are likely to be in the same network community if one of them is named as a social contact by the other’s social contacts. We find that the friend of a friend is 3.0 times (99.5 percent CI: 2.9, 3.1) more likely to be to be in the same community than expected due to chance. However, the numbers are much lower for any set of relations containing a negative tie. The friend of an antagonist is only 1.5 times (99.5 percent CI: 1.4, 1.5) more likely than by chance to be in the same community, the antagonist of a friend is 1.5 times (99.5 percent CI: 1.5, 1.6) more likely, and the antagonist of an antagonist is 1.8 times (99.5 percent CI: 1.7, 1.9) more likely. This may help to explain why the only two-degree relationship that is associated with a friendship is the friend of a friend. We find our friends and our antagonists in the same network communities.

**Figure 6:**
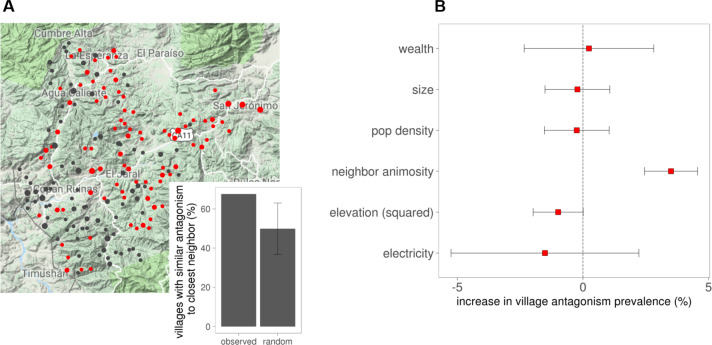
Analysis of village-level antagonism prevalence. (A) Topographical map of villages by size and prevalence of antagonism (N = 176 villages). Colors represent the prevalence of antagonism (ratio of negative ties to positive-plus-negative ties; red = higher than median, and black = lower than median; namely, 12.8 percent). Point sizes correspond to village size (from smallest to largest: *<*150 people, 150–300 people, and *>*300 people). The inset shows the observed percentage of villages (67.6 percent) that have the same antagonism prevalence (low or high) as their nearest geographic neighbor (“as the crow flies”) compared to a simulation in which antagonism levels are randomly permuted among villages (N = 1,000 simulations); the black bar represents a 99.5 percent confidence interval. There is significant geographic clustering of antagonism prevalence (*p <* 0.005). (B) Percentage increase in antagonism prevalence (*x* axis) based on bivariate models of village-level variables indicating how much a one–standard-deviation change in each of the variables shown (*y* axis) is associated with an increased prevalence of antagonism from an ordinary least squares (OLS) estimate. A one-unit change is shown for factor variables (e.g., wealth and electricity). The estimate for elevation includes a squared term, and the change is calculated from an elevation centered on the maximum of the parabola, so only the effect of the squared term is shown. Black lines around estimates indicate 99.5 percent confidence intervals (see the online supplement).

Finally, whereas the mean percentage of negative ties (i.e., the number of antagonistic ties divided by the number of friendship ties) was 16.0 percent, the percentage of antagonism varied substantially across villages, from 1.1 percent to 40.0 percent. A topographical map of the villages is shown in Figure 6A (colors represent the prevalence of animosity: red = higher than median, black = lower than median; point sizes correspond to village size: smallest = *<*150 people, medium = 150–300 people, and large = *>*300 people). Most villages (68 percent) have the same prevalence level of animosity (defined as the ratio of negative ties divided by all ties) as their nearest neighbor, which is significantly more than due to chance (*p <* 0.005). In 1,000 randomly permuted networks, on average, 50 percent of villages had the same level of animosity (99.5 percent CI: 37.2 percent, 63.3 percent). A set of models evaluating village-level variables (such as population, population density, elevation, wealth, infrastructure, and the prevalence of antagonism in the closest neighboring village; Figure 6B and online supplement Tables S15–S22) shows that only nearest-village antagonism has a statistically significant association with the level of antagonism (*p <* 0.005), including in multivariate models. However, there is some evidence that villages at an intermediate elevation have the highest prevalence of antagonism, even after controlling for the antagonism of the closest neighboring village (*p <* 0.05).

## Discussion

Real social networks manifest friendship and antagonism concurrently. People can form positive and negative (or no) sentiments regarding people with whom they interact, and antagonistic relations are important determinants of individual and group behavior (Labianca and Brass 2006). Villages in the developing world (such as our setting in Honduras) offer an especially appealing natural laboratory to evaluate the structure of negative ties because they are relatively closed social systems where people cannot easily avoid others whom they may come to dislike. In this setting, we find that apparently similar underlying social processes (based on sustained and repeated interactions) result in positive and negative ties having generally similar structural properties, qualitatively speaking. Nevertheless, negative ties are also different; quantitatively, they are less common, somewhat less homophilous, and less often reciprocated.

In addition, we find that people with more friends also have more antagonists. And we find substantial empirical support, on a large scale, for certain (but not all) aspects of balance theory long theorized to affect triadic social interactions and the networks that they give rise to. People tend to have negative ties with their friends’ antagonists and their antagonists’ friends, and they avoid negative ties with their friends’ friends. People also tend to form numerically more negative ties with members of other network communities rather than members of their own, which is in keeping with theories about social cohesion (Kawachi and Berkman 2000). These phenomena suggest that negative ties play a role in the structure of networks otherwise based solely on positive ties.

Despite their possible implications, less work has been done to ascertain antagonistic-relationship frequency in real social networks, and estimates can vary according to how the ties are ascertained (White 1961; Labianca and Brass 2006, Leskovec, Huttenlocher, and Kleinberg 2010; Szell et al. 2010; Offer and Fischer 2017). We find that negative ties are not uncommon (16.0 percent on average, expressed as the fraction of negative ties to positive ties). We also find that this percentage varied widely across villages (from 1.1 percent to 40 percent). The prevalence of antagonistic ties seems to vary geographically, with nearby villages showing similar levels of antagonism. Assessing how the frequency and, possibly, structural location of negative ties varies according to environmental circumstances or according to the weight of the ties (e.g., whether someone “hates” or merely “does not get along with” another person) is an important area for future work.

Social networks based on positive ties—from diverse settings ranging from foragers (Apicella et al. 2012) to agropastoralists (Glowacki et al. 2016) to villagers (Banerjee et al. 2013; Kim et al. 2015) to city dwellers (Barabasi and Albert 1999; Onnela et al. 2007; Palla, Barabasi, and Vicsek 2007; Bond et al. 2012)—share structural similarities. This similarity has led to the proposition that their formation depends on fundamental properties of humans and their interactions and that it is shaped by natural selection in ways that affect a diverse set of human social behaviors and our evolved psychology (Tooby and Cosmides 1996; Hruschka and Henrich 2006; Christakis and Fowler 2014). Evolutionary forces may thus also have shaped how we think and feel about, and how we pick, our enemies. Individuals and groups might possibly benefit from these properties if, for instance, the community structure of the networks around individuals is relevant to their outcomes.

In principle, negative ties could serve a valuable purpose in social networks, just as they do in situations far beyond social networks; for instance, they are critical component for the proper functioning and synchronization of brain patterns (Bargmann and Marder 2013; Isakov and Mahadevan 2014) and in deep learning algorithms (Marblestone, Wayne, and Kording 2016). One purpose might be to play a role in structuring networks for some sort of social optimality, such as that involving cooperation. A shared antagonist may not only foster cohesion among members of groups as a whole but also among pairs of individuals. For instance, A may be more likely to cooperate with B if they have C as a common enemy rather than as a common friend. Prior experimental work has shown that people cut ties to those who take advantage of them in cooperative interactions and that this kind of “decentralized ostracism” helps stabilize cooperation (Rand, Arbesman, and Christakis 2011). Possibly, having antagonists could work analogously, stabilizing cooperation. Future experiments could shed light on this. Furthermore, future work assessing the relationship of negative ties to subsequent village-level collective action (such as the maintenance of property held in common), exploiting longitudinal data, would also shed light on why some developing-world villages fare better than others during development initiatives; possibly, an in-between level of antagonism might be optimal in this regard.

The theorized psychological mechanism underlying balance theory is cognitive dissonance: the state of mental stress a person experiences while holding two conflicting beliefs, ideas, or values. The theory postulates that it imposes too high a cognitive load to see both the good and bad in others, and so people are dichotomized into two categories (Festinger 1962). Balance may be preferred by humans simply because it is easier to encode balanced social relations into memory (Brashears and Brashears 2016). Structural balance may therefore reflect the social-psychological interdependencies that strain cognitive consistency (Heider 1946; Cartwright and Harary 1956; Rawlings and Friedkin 2017). Our work comports with these theories of the evolved psychology of antagonistic ties. Still, we did not ascertain actual mechanisms for ties forming or breaking nor subjects’ beliefs about notions of balance. Moreover, we did not have longitudinal information about the network. The empirical dynamics of signed social networks is another important area for future work.

Social conflict is ubiquitous, and not just in rural villages. Our networks and our psychology reflect this. But antagonistic ties might actually be constructive, playing a crucial role in the structure of social relations in human groups.

## Methods

### Data

Data were collected in the Copan province of western Honduras in 2016. The sample population included only people older than age 12 years in 176 villages. We were able to census 30,820 of these people in the target villages (94 percent), and of these, 24,696 (81 percent) participated in our full survey and in detailed geographic and sociocentric mapping. We used our custom Trellis software platform (available at trellis.yale.edu) and tablet-based surveys administered face to face in order to collect detailed social-network information with three name generators to determine (often overlapping) positive ties (“Who do you spend your free time with?” “Who is your closest friend?” and “Who do you discuss personal matters with?”) and one name generator for negative ties (“Who are the people with whom you do not get along well?”). People could, and sometimes did, name their siblings for these ties (10.2 percent and 0.6 percent of all positive and negative ties, respectively). Because the villages were small and were sociocentrically mapped, unnamed individuals with whom the respondent had no particular relationship could be labelled as strangers in the sense that they were neither friends nor antagonists. By design, all ties were discerned within each village (in any case, only a minority of meaningful ties were outside the villages).

There are various questions that could be used to tap into different sorts or levels of antagonism, such as “hatred” or “annoyance,” but we decided that general negative affect is most epitomized by “do not like” (in Spanish) and that this would also be more frequent than many alternatives. One problem with self-reported antagonistic ties in general is that people display a reticence to speak negatively of other people in their communities because of social desirability (see the online supplement). And although observing friendship via proxies, such as spending time together, is feasible, it is harder to discern the difference between enemies and strangers without directly asking subjects.

The social networks were drawn with Cytoscape (Shannon et al. 2003) using a force-directed layout algorithm. Edge colors indicate relationship status (gray = friends; red = antagonists). Arrows indicate the nomination direction.

Approval for this study was obtained from the Ministry of Health in Honduras and from the Yale University Institutional Review Board. Informed consent was obtained from all participants. Full code and a sample of data for 11 villages are available on our lab website.

### Heterogenous Triad Census and Social Rules

We performed an exhaustive search to determine the full triad census on a network with directed ties (both positive and negative). We also calculated the total number of times each of the following social rules was true for each ego (the number of triads of the form “the *X* of my *Y* is my *Z*,” where *X*, *Y*, *Z ∈ { friend*, *antagonist*, *stranger}*). See the online supplement for additional details.

### Comparisons to Empirically Calibrated Random Networks

We generated random worlds wherein each world consisted of the full set of 176 permuted village networks. Each village network had random rewiring of friend and antagonistic ties, ensuring that the total number of each tie type remained constant, which is the same as in the observed networks. Additionally, we tested balance with exponential random graph models (ERGMs) using XPNet (Wang, Robins, and Pattison 2009) and found qualitatively similar results, although this method presented significant convergence issues.

### Statistical Modeling

To explore associations between the structure of networks with negative ties, we fit generalized linear models with a logit link function to the data in order to estimate the association between the existence of social ties in a village and various network characteristics. The basic model at the dyad level is

$$\begin{array}{c} E[{ϒ}_{ego,alter}]=\mu =g^{-1}\left(\alpha +\beta_{1}x_{ego}+\beta_{2}x_{alter}+\beta_{3}\left(-|x_{ego}-x_{alter}|\right)+\gamma_{v}\right)\\ g\left(\mu \right)=\ln\left(\frac{\mu }{1-\mu }\right) \end{array}$$

Here, *Y*_*ego,alter*_ is 1 if ego *i* nominates alter *j* as a friend (antagonist), ***x_ego_*** is a vector of characteristics for ego, ***xalter*** is a vector of characteristics for alter, and *|**x_ego_−xalter** |* is the absolute difference in characteristics between the ego and alter. The coefficients ***β*****_1_** and ***β*****_2_** indicate how much a unit change in the independent variable is associated with an increase in the log odds of the existence of a tie, and ***β*****_3_** indicates how much homophily (ego–alter similarity) on the independent variable is associated with the corresponding increase in the log odds. The signs are chosen such that higher values correspond to people with social ties that are more similar to one another than people without social ties. To account for potential differences attributable to the particular village where the ego and alter live, we include village fixed effects (*γ_v_*). Here, *α* is a constant, which we drop when fixed effects are included.

To compare friendly and antagonistic ties, we measured their propensity to exist between ego–alter pairs. Measured personal characteristics (including ego and alter age, sex, whether they identify with a religion, indigenous status, a dichotomized measure of household wealth, and a dichotomized measure of health) and network characteristics (in-degree, out-degree, reciprocity, and the number of common friends and/or antagonists) were tested for association with the probability of a positive or negative tie in bivariate models without controls (see online supplement Table S1 for summary statistics). These models include a measurement for the ego, alter, and similarity between them (see online supplement Tables S2–S11). Throughout, we used a significance value of *p <* 0.005 (Benjamin et al. 2018).

To make comparisons, we used the models to estimate the percentage increases in the odds of forming a social tie associated with changing the independent variable by one unit and holding all other variables constant.

To explore what village-level characteristics may contribute to the prevalence of negative ties, we fit linear regressions to the data. The basic model at the village level is

$$E[{ϒ}_{i}]=\alpha +\beta x_{i},$$

where the dependent variable *Y_i_* is the prevalence of antagonism (the ratio of the number of negative ties to the number of all ties [i.e., the sum of positive and negative ties]) in village *i*, ***x_i_*** is a vector of village characteristics, and ***β*** is a vector of coefficients that indicate the degree of association with each characteristic. Measured village-level characteristics are village size, population density (measured as the average distance between households), village elevation, a wealth index, level of infrastructure (measured as whether there is electricity), and prevalence of animosity in the closest neighboring village. Again, we used a significance value of *p <* 0.005. To make comparisons at the village level, we used the models to estimate the increase in the prevalence of animosity associated with changing the independent variable by one standard deviation (one unit for factor variables) and holding all other variables constant.
