# Supplementary material for: The Structure of Negative Social Ties in Rural Village Networks
Source: Sociol Sci. 2019 Mar 6;6:197–218. doi: 10.15195/v6.a8 (PMC7340493; doi:10.15195/v6.a8)
Supplement: Supplementary file 1 [file SS-2019-V6-A8-s1.pdf]

sociological science

Supplement to:

Isakov, Alexander, James H. Fowler, Edoardo M. Airoldi, and Nicholas A. Christakis. 2019. “The Structure of Negative Social Ties in Rural Village Networks.” Sociological Science 6: 197-218.

Online Supplement

The Structure of Negative Social Ties in Rural Village Networks

Alexander Isakov,[1,2] James H. Fowler,[3,4] Edoardo M. Airoldi,[5,6] Nicholas A. Christakis[1,2,7,8*]

[1] *Yale Institute for Network Science, Yale University, New Haven, CT 06520, USA.*
[2] *Department of Sociology, Yale University, New Haven, CT 06520, USA.*
[3] *Department of Medicine, University of California, San Diego, CA 92103, USA.*
[4] *Political Science Department, University of California, San Diego, CA 92103, USA.*
[5] *Department of Statistical Science, Fox School of Business, Temple University, Philadelphia, PA 19122, USA.*
[6] *Department of Statistics and Institute for Quantitative Social Sciences, Harvard University, Cambridge, MA 02138, USA.*
[7] *Department of Ecology and Evolutionary Biology, Yale University, New Haven, CT 06520, USA.*
[8] *Department of Statistics and Data Science, Yale University, New Haven, CT 06520, USA.*

*To whom correspondence should be addressed. Author e-mail: nicholas.christakis@yale.edu

Supplementary Materials and Methods

Characterizing Unique Triads in Directed Graphs with Friend and Antagonist Ties

We developed the "Heterogenous Triad Census" as a complete triad census on directed graphs with heterogenous edges (i.e., a unique categorization of triads). Each directed edge can have one of two colors (corresponding to "friend" and "antagonist"). Define a network G to be in the same isomorphism class as a network G' if it is the same up to node relabeling. *A priori*, there are $3^6 = 729$ potentially distinct networks with 3 nodes, no self-loops (that is, with 0 on the diagonal of the corresponding adjacency matrix), and 3 possible entries in the each of the other 6 slots (0 = no edge, -1 = antagonist edge, 1 = friend edge). An exhaustive search gives an explicit splitting into isomorphism classes. Thus, we discern 138 unique triad classes.

For simplicity of reference, we propose labeling the triad classes with the following scheme. Every class has a name $\boldsymbol{B_i F_j E_k U_l S_m}$, where "B" stands for "Blank," "F" stands for

"Friend," "E" stands for "Antagonist" ("Enemy"), "U" stands for "Unsymmetrizable," and "S" stands for "Symmetry," and i, j, k, l, m are integers defined as follows. Let $\boldsymbol{A}$ be the adjacency matrix corresponding to the triple and call the nodes α, β, γ. Then,

- i is the number of pairs of three vertices that have no ties in either direction ($\boldsymbol{A}_{xy} = \boldsymbol{A}_{yx} = 0$ where $x, y \in \{\alpha, \beta, \gamma\}$ and $x \neq y$ since we do not allow self-loops);
- j is the number of pairs of friend ties that either are already symmetric or can be symmetrized into a friend pair (either $\boldsymbol{A}_{xy} = \boldsymbol{A}_{yx} = 1$, or $\boldsymbol{A}_{xy} = 1$ and $\boldsymbol{A}_{yx} = 0$);
- k is the number of pairs of antagonist ties that either are already symmetric or can be symmetrized into an antagonist pair (either $\boldsymbol{A}_{xy} = \boldsymbol{A}_{yx} = -1$, or $\boldsymbol{A}_{xy} = -1$ and $\boldsymbol{A}_{yx} = 0$)
- l is the number of pairs of friend/antagonist ties that cannot be symmetrized ($\boldsymbol{A}_{xy} = 1$ and $\boldsymbol{A}_{yx} = -1$);
- m is an index for symmetry breaking (starting from 0) for classes that would be the same when friend or antagonist ties are symmetrized.

For example, the "empty triad" has a Class Name of $B_3F_0E_0U_0S_0$, since all three pairs of vertices have no connections between them in either direction. The full census, with figures, class names, and total observed counts of each class across all villages, is provided in Supplementary Table S23. A summary of sociologically relevant triads is provided in Table S13. These results are applicable to any directed network with multiple tie types, including those found in neurobiology (where connections can be excitatory or inhibitory) and social science (including multiplex networks).

Network Parameters

Reciprocity is measured as the probability that the edge from ego to alter exists if the edge from alter to ego exists. That is, we count the number of times a directed edge is reciprocated and divide by the number of edges. We report these measures separately for friend connections and antagonist connections across the whole population. In other words, if $A^{V,S}$ is the adjacency matrix for ties of type S (S ∈ {Friend, Antagonist}) ties for village V, then:

$$Reciprocity_S = \frac{\sum_V \left(\sum_{i,j} A_{ij}^{V,S} \left(A_{ij}^{V,S}\right)^T\right)}{\sum_V \left(\sum_{i,j} A_{ij}^{V,S}\right)}, \mathrm{S} \in \{\mathrm{Friend}, \mathrm{Antagonist}\}$$

Transitivity (the likelihood that two of a person's friends (antagonists) are themselves friends (antagonists)) is calculated as a global network parameter, i.e., the ratio of connected triples to the total number of possible connected triples in an undirected network (e.g., in a village). We report this standard measure separately for the friendship and antagonism networks:

$$Transitivity_S = \frac{\sum_V Tr((A^{V,S})^3)}{\sum_V ((\sum_{i,j} (A_{ij}^{V,S})^2) - Tr((A^{V,S})^2))}, \mathrm{S} \in \{\mathrm{Friend}, \mathrm{Antagonist}\}$$

Community Detection for Negative Tie Structure

To evaluate the structural location of negative ties in higher-order network features (network communities), we first create subsets of the village networks that included only positive ties. We partition this subnetwork into communities, reinsert negative ties, and compute the ratio of negative ties between communities compared to within communities. To assess the sensitivity of the results to the community detection method used, we employed three distinct methods and found similar results: a fast-greedy community-detection algorithm (where the ratio is 3.0, 99.5%

CI: 5.1, 5.9), the Louvain method (4.3, 99.5% CI: 7.3, 8.7), and the Girvan-Newman method of edge betweenness (4.5, 99.5% CI: 12.2, 15.9). In all cases, there are relatively fewer negative ties between communities than due to random chance alone.

Basic Logistic Regression Models

Table S1 shows summary statistics. Tables S2-S11 show bivariate logistic regressions (GLMs) across all possible dyads in the network. Table S12 shows a multivariate model with all covariates, where the dependent variable is 1 if person i names person j as a social tie (we analyze friends and antagonists separately), and 0 otherwise. For all models, we used village fixed effects to remove the impact of village-specific characteristics. All p-values are given to three significant digits (those given as 0.000 are $\ll 0.001$). The independent variables in the basic model include both "personal" and "social" variables. Personal variables are individual characteristics: sex, age, whether or not they are indigenous, whether or not they identify with a religion, their dichotomized wealth level ("wealthy" if they responded 4 or higher on a summary measure of wealth and "not wealthy" otherwise), and their dichotomized health levels ("healthy" if they responded 4 or higher on a summary measure of health and "not healthy" otherwise). Social variables include reciprocity, number of friends, number of antagonists, and amount of overlap (number of co-nominated friends/antagonists). The coefficients indicate the log-odds of the existence of the corresponding type of tie.

Comparing Second Moments of Friendship and Antagonism

We used a stringent test to determine whether the second moment of friend and antagonist ties is significantly different. First, we standardized the distribution of means by removing friend ties at random such that the total number of friend and antagonist ties within

each village was equal. Then, we calculated the standard deviation of the degree distribution for friend and antagonist ties separately. We used a paired t-test at the 99.5% confidence level to conclude that the second moment of friend ties is not equal to that of antagonist ties ($P \ll 0.005$). See Figure S1 and Table S14 for results from a simple linear regression.

Basic Village-Level Regression Models

To study the prevalence of animosity (number of antagonist ties divided by number of friend and antagonist ties), we used bivariate OLS regressions across all villages in the sample. The results are shown in Tables S15-S22. All p-values are given to three significant digits (those given as 0.000 are $\ll 0.001$). The independent variables in the basic model are village size, population density (measured as the average distance between households), village elevation (and elevation squared), a wealth index, whether or not there is electricity (level of infrastructure), and the prevalence of animosity in the closest neighboring village ("as the crow flies", found using the Haversine formula from the village center). The coefficients indicate how much a unit change in the independent variable is associated with an increase in the prevalence of animosity in a village.

Supplementary Figures

Supplementary Figure S1

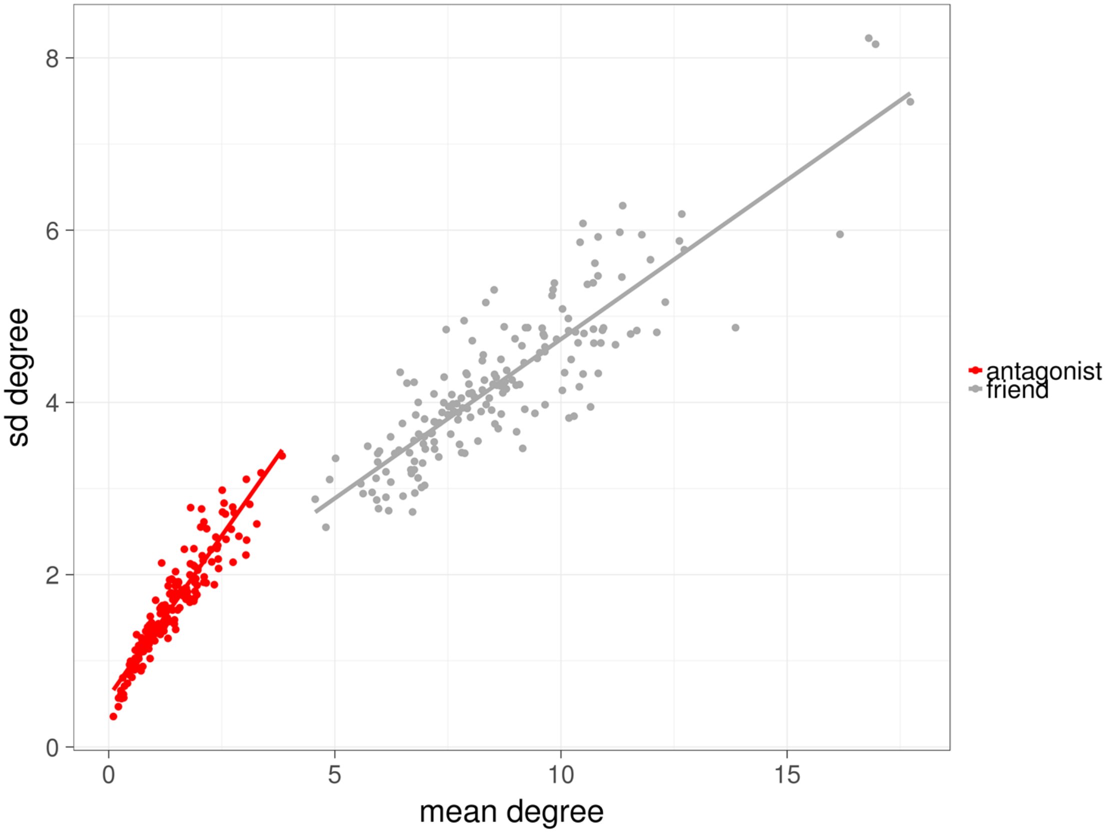

Figure S1. Mean vs. standard deviation for friend and antagonistic ties. Conditional on the mean, the standard deviation for enemies is approximately twice as large as for friends, suggesting that antagonistic relationships are created in a less structured social context than friendship relationships.

Supplementary Tables

Supplementary Table S1: Summary Statistics.

Variable	*Mean*	*SD*	*Min*	*Max*
Male	0.424	0.494	0	1
Age	32.843	17.168	11	93
Religiosity	0.836	0.370	0	1
Indigenous Status	0.117	0.321	0	1
Income Level (High)	0.388	0.487	0	1
General Health Status (Healthy)	0.201	0.401	0	1
In-Degree (Friends)	4.259	3.448	0	34
Out-Degree (Friends)	4.259	2.561	0	29
In-Degree (Antagonists)	0.666	1.272	0	25
Out-Degree (Antagonists)	0.666	1.166	0	16

Supplementary Table S2: Logistic Regression of Social Ties on Reciprocity

	Dependent Variable: Ego Is Antagonists with Alter			*Dependent Variable: Ego Is Friends with Alter*		
	Coef.	*S.E.*	*P*	*Coef.*	*S.E.*	*P*
Reciprocal Tie Exists	**2.181**	**0.037**	**0.000**	**3.238**	**0.008**	**0.000**
Village Fixed Effects	YES			YES		
N	4698552			4698552		

Logistic regression presence of social tie from ego to alter on the existence of tie reciprocity. Model includes village fixed effects (not shown).

Supplementary Table S3: Logistic Regression of Social Ties on Sex

	Dependent Variable: Ego Is Antagonists with Alter			*Dependent Variable: Ego Is Friends with Alter*		
	Coef.	*S.E.*	*P*	*Coef.*	*S.E.*	*P*
Ego Male	**-0.325**	**0.021**	**0.000**	**0.107**	**0.007**	**0.000**
Alter Male	**0.176**	**0.021**	**0.000**	**0.135**	**0.007**	**0.000**
Ego and Alter are Same Sex	**1.484**	**0.021**	**0.000**	**0.942**	**0.007**	**0.000**
Village Fixed Effects		YES			YES	
N		4698552			4698552	

Logistic regression of presence of social tie from ego to alter on ego and alter sex. Model includes village fixed effects (not shown).

Supplementary Table S4: Logistic Regression of Social Ties on Age

	Dependent Variable: *Ego Is Antagonists with Alter*			*Dependent Variable:* *Ego Is Friends with Alter*		
	Coef.	*S.E.*	*P*	*Coef.*	*S.E.*	*P*
Ego Age	**-0.007**	**0.0006**	**0.000**	**0.006**	**0.0002**	**0.000**
Alter Age	**0.030**	**0.0006**	**0.000**	**0.020**	**0.002**	**0.000**
Ego-Alter Age Similarity	**0.038**	**0.0007**	**0.000**	**0.031**	**0.003**	**0.000**
Village Fixed Effects		YES			YES	
N		4698552			4698552	

Logistic regression of presence of social tie from ego to alter on ego and alter age. Model includes village fixed effects (not shown).

Supplementary Table S5: Logistic Regression of Social Ties on Religion

	Dependent Variable: Ego Is Antagonists with Alter			*Dependent Variable: Ego Is Friends with Alter*		
	Coef.	*S.E.*	*P*	*Coef.*	*S.E.*	*P*
Ego Religious	**0.080**	**0.028**	**0.005**	**-0.150**	**0.011**	**0.000**
Alter Religious	**-0.102**	**0.028**	**0.000**	**-0.032**	**0.011**	**0.002**
Ego-Alter Same Religious Status	**0.130**	**0.028**	**0.000**	**0.399**	**0.011**	**0.000**
Village Fixed Effects		YES			YES	
N		4698552			4698552	

Logistic regression of presence of social tie from ego to alter on ego and alter identifying with a religion. Model includes village fixed effects (not shown).

Supplementary Table S6: Logistic Regression of Social Ties on Health

	Dependent Variable: *Ego Is Antagonists with Alter*			*Dependent Variable:* *Ego Is Friends with Alter*		
	Coef.	*S.E.*	*P*	*Coef.*	*S.E.*	*P*
Ego Healthy	**-0.113**	**0.027**	**0.000**	**0.084**	**0.010**	**0.000**
Alter Healthy	**-0.079**	**0.027**	**0.003**	0.021	0.010	0.033
Ego-Alter Same Health Status	**0.131**	**0.027**	**0.000**	**0.151**	**0.010**	**0.000**
Village Fixed Effects		YES			YES	
N		4697884			4697884	

Logistic regression of presence of social tie from ego to alter on ego and alter health status. Model includes village fixed effects (not shown).

Supplementary Table S7: Logistic Regression of Social Ties on Wealth

	Dependent Variable: Ego Is Antagonists with Alter			*Dependent Variable: Ego Is Friends with Alter*		
	Coef.	*S.E.*	*P*	*Coef.*	*S.E.*	*P*
Ego Wealthy	**-0.101**	**0.018**	**0.000**	**0.027**	**0.008**	**0.000**
Alter Wealthy	**0.119**	**0.018**	**0.000**	**0.279**	**0.008**	**0.000**
Ego-Alter Same Wealth Status	0.036	0.017	0.041	**0.628**	**0.007**	**0.000**
Village Fixed Effects		YES			YES	
N		4609496			4609496	

Logistic regression of presence of social tie from ego to alter on ego and alter wealth status. Model includes village fixed effects (not shown).

Supplementary Table S8: Logistic Regression of Social Ties on Indigenous Status

	Dependent Variable: Ego Is Antagonists with Alter			*Dependent Variable: Ego Is Friends with Alter*		
	Coef.	*S.E.*	*P*	*Coef.*	*S.E.*	*P*
Ego is Indigenous	**0.266**	**0.036**	**0.000**	**0.166**	**0.014**	**0.000**
Alter is Indigenous	**0.198**	**0.036**	**0.000**	**0.244**	**0.014**	**0.000**
Ego-Alter Same Indigenous Status	0.080	0.032	0.013	**0.315**	**0.012**	**0.000**
Village Fixed Effects		YES			YES	
N		4694224			4694224	

Logistic regression of presence of social tie from ego to alter on whether ego and alter are indigenous. Model includes village fixed effects (not shown).

Supplementary Table S9: Logistic Regression of Social Ties on Friend on Out-Degree

	Dependent Variable: Ego Is Antagonists with Alter			*Dependent Variable: Ego Is Friends with Alter*		
	Coef.	*S.E.*	*P*	*Coef.*	*S.E.*	*P*
Ego Friend Out-Degree	**0.103**	**0.003**	**0.000**	**0.225**	**0.001**	**0.000**
Alter Friend Out-Degree	0.008	0.003	0.012	**0.050**	**0.001**	**0.000**
Ego-Alter Friend Out-Degree Similarity	**0.013**	**0.004**	**0.001**	**0.082**	**0.002**	**0.000**
Village Fixed Effects		YES			YES	
N		4698552			4698552	

Logistic regression of presence of social tie from ego to alter on ego and alter friend in-degree. Model includes village fixed effects (not shown).

Supplementary Table S10: Logistic Regression of Social Ties on Antagonist Out-Degree

	Dependent Variable: Ego Is Antagonists with Alter			*Dependent Variable: Ego Is Friends with Alter*		
	Coef.	*S.E.*	*P*	*Coef.*	*S.E.*	*P*
Ego Antagonist Out-Degree	**0.756**	**0.008**	**0.000**	**0.117**	**0.003**	**0.000**
Alter Antagonist Out-Degree	0.008	0.007	0.311	**0.020**	**0.003**	**0.000**
Ego-Alter Antagonist Out-Degree Similarity	**0.243**	**0.008**	**0.000**	**0.078**	**0.004**	**0.000**
Village Fixed Effects		YES			YES	
N		4698552			4698552	

Logistic regression of presence of social tie from ego to alter on ego and alter antagonist in-degree. Model includes village fixed effects (not shown).

Supplementary Table S11: Logistic Regression of Social Ties on Friend and Antagonist Co-nominations

	Dependent Variable: *Ego Is Antagonists with Alter*			*Dependent Variable:* *Ego Is Friends with Alter*		
	Coef.	*S.E.*	*P*	*Coef.*	*S.E.*	*P*
Number of Friends Co-nominated	**0.198**	**0.011**	**0.000**	**1.180**	**0.004**	**0.000**
Number of Antagonists Co-nominated	**0.527**	**0.035**	**0.000**	**0.633**	**0.018**	**0.000**
Village Fixed Effects		YES			YES	
N		4698552			4698552	

Logistic regression of presence of social tie from ego to alter on the number of friends and antagonists co-nominated by ego and alter. Model includes village fixed effects (not shown).

Supplementary Table S12: Logistic Regression of Social Ties on All Personal and Social Covariates

	Dependent Variable: Ego Is Antagonists with Alter			*Dependent Variable: Ego Is Friends with Alter*		
	Coef.	*S.E.*	*P*	*Coef.*	*S.E.*	*P*
Reciprocal Tie Exists	**1.920**	**0.042**	**0.000**	**2.821**	**0.010**	**0.000**
Ego Male	**-0.291**	**0.022**	**0.000**	**0.078**	**0.008**	**0.000**
Alter Male	**0.243**	**0.022**	**0.000**	**0.049**	**0.008**	**0.000**
Ego and Alter are Same Sex	**1.489**	**0.022**	**0.000**	**0.764**	**0.008**	**0.000**
Ego Age	**-0.008**	**0.001**	**0.000**	**0.003**	**0.000**	**0.000**
Alter Age	**0.017**	**0.001**	**0.000**	**0.008**	**0.000**	**0.000**
Ego-Alter Age Similarity	**0.035**	**0.001**	**0.000**	**0.023**	**0.000**	**0.000**
Ego Religious	**0.086**	**0.030**	**0.004**	**-0.074**	**0.012**	**0.000**
Alter Religious	**-0.126**	**0.030**	**0.000**	**-0.153**	**0.012**	**0.000**
Ego-Alter Same Religious Status	0.028	0.029	0.327	**0.222**	**0.012**	**0.000**
Ego Healthy	**-0.154**	**0.028**	**0.000**	**0.062**	**0.011**	**0.000**
Alter Healthy	**0.101**	**0.028**	**0.000**	**0.058**	**0.011**	**0.000**
Ego-Alter Same Health Status	0.075	0.027	0.006	**0.061**	**0.011**	**0.000**
Ego Wealthy	**-0.055**	**0.019**	**0.003**	-0.023	0.008	0.007
Alter Wealthy	0.049	0.019	0.010	**0.170**	**0.008**	**0.000**
Ego-Alter Same Wealth Status	0.020	0.018	0.257	**0.485**	**0.008**	**0.000**
Ego is Indigenous	**0.299**	**0.036**	**0.000**	**0.068**	**0.015**	**0.000**
Alter is Indigenous	0.077	0.037	0.039	**0.078**	**0.016**	**0.000**
Ego-Alter Same Indigenous Status	0.021	0.033	0.532	**0.158**	**0.014**	**0.000**

Ego Friend In-Degree	**-0.014**	**0.003**	**0.000**	**-0.072**	**0.001**	**0.000**
Alter Friend In-Degree	**0.030**	**0.003**	**0.000**	**0.223**	**0.001**	**0.000**
Ego-Alter Friend In-Degree Similarity	0.004	0.003	0.227	**0.090**	**0.001**	**0.000**
Ego Antagonist In-Degree	-0.004	0.007	0.575	0.007	0.004	0.045
Alter Antagonist In-Degree	**0.554**	**0.007**	**0.000**	**0.047**	**0.004**	**0.000**
Ego-Alter Antagonist In-Degree Similarity	**0.240**	**0.007**	**0.000**	**0.048**	**0.004**	**0.000**
Number of Friends Co-nominated	**0.075**	**0.013**	**0.000**	**0.434**	**0.021**	**0.000**
Number of Antagonists Co-nominated	**0.388**	**0.039**	**0.000**	**0.878**	**0.004**	**0.000**
Village Fixed Effects		YES			YES	
N		4604534			4604534	

Logistic regression of presence of social tie from ego to alter on all personal and social characteristics. Model includes village fixed effects (not shown).

Supplementary Table S13. Summary of counts of triadic relationships

Relationship Type		Real Observation Counts
The Friend of my Friend is my…	Friend	96,544
	Stranger	371,236
	Antagonist	4,159
The Antagonist of my Friend is my…	Friend	3,966
	Stranger	63,315
	Antagonist	3,601
The Friend of my Antagonist is my…	Friend	4,889
	Stranger	63,513
	Antagonist	5,370
The Antagonist of my Antagonist is my…	Friend	1,630
	Stranger	12,866
	Antagonist	577

Supplementary Table S14: OLS Regression of Second Moment of Degree on Mean Degree

	Dependent Variable: Standard Deviation of Degree		
	Coef.	*S.E.*	*P*
Intercept	**1.038**	**0.174**	**0.000**
Mean Degree	**-0.370**	**0.021**	**0.000**
Antagonist Tie Network Indicator	**-0.455**	**0.171**	**0.008**
Antagonist Tie Network Indicator*Mean Degree	**0.378**	**0.029**	**0.000**
Village Fixed Effects		NO	
Adjusted R^2		0.944	
N		352	

OLS regression of second moment of the friend and antagonist degree distributions, with multi-way clustering of standard errors on village.

Supplementary Table S15: OLS Regression of Village Level Antagonism Prevalence on Village Size

	Dependent Variable: *Percent of Antagonistic Ties to All Social Ties*		
	Coef.	*S.E.*	*P*
Intercept	**13.730**	**0.088**	**0.000**
Village Size	-0.002	0.005	0.633
Adjusted R^2		0.001	
N		176	

OLS regression of antagonism prevalence on village size.

Supplementary Table S16: OLS Regression of Village Level Antagonism Prevalence on Village Population Density

	Dependent Variable: *Percent of Antagonistic Ties to All Social Ties*		
	Coef.	*S.E.*	*P*
Intercept	**13.909**	**1.111**	**0.000**
Population Density	-0.001	0.002	0.594
Adjusted R^2		0.001	
N		176	

OLS regression of antagonism prevalence on village population density, measured as average geographic distance between households ("as the crow flies").

Supplementary Table S17: OLS Regression of Village Level Antagonism Prevalence on Elevation

	Dependent Variable: Percent of Antagonistic Ties to All Social Ties		
	Coef.	*S.E.*	*P*
Intercept	-16.920	9.577	0.078
Elevation	**0.064**	**0.021**	**0.003**
$Elevation^2$	**-0.00003**	**0.00001**	**0.006**
Adjusted R^2		0.051	
N		176	

OLS regression of antagonism prevalence on elevation.

Supplementary Table S18: OLS Regression of Village Level Antagonism Prevalence on Village Size

	Dependent Variable: Percent of Antagonistic Ties to All Social Ties		
	Coef.	*S.E.*	*P*
Intercept	**12.678**	**2.684**	**0.000**
Wealth Index	0.240	0.919	0.794
Adjusted R^2		0.000	
N		176	

OLS regression of antagonism prevalence on village wealth index.

Supplementary Table S19: OLS Regression of Village Level Antagonism Prevalence on Electricity

	Dependent Variable: *Percent of Antagonistic Ties to All Social Ties*		
	Coef.	*S.E.*	*P*
Intercept	**14.673**	**1.237**	**0.000**
Village Has Electricity	-1.511	1.331	0.258
Adjusted R^2		0.002	
N		176	

OLS regression of antagonism prevalence on village infrastructure (whether the village has electricity).

Supplementary Table S20: OLS Regression of Village Level Antagonism Prevalence on Closest Neighboring Village Antagonism Prevalence

	Dependent Variable: *Percent of Antagonistic Ties to All Social Ties*		
	Coef.	*S.E.*	*P*
Intercept	**5.959**	**0.878**	**0.000**
Closest Village Animosity	**56.450**	**6.049**	**0.000**
Adjusted R^2		0.330	
N		176	

OLS regression of antagonism prevalence on the antagonism prevalence of the geographically closest neighboring village (“as the crow flies”).

Supplementary Table S21: OLS Regression of Village Level Antagonism Prevalence on Village Size

	Dependent Variable: *Percent of Antagonistic Ties to All Social Ties*		
	Coef.	*S.E.*	*P*
Intercept	**13.730**	**0.088**	**0.000**
Village Size	-0.002	0.005	0.633
Adjusted R^2		0.001	
N		176	

OLS regression of antagonism prevalence on village size.

Supplementary Table S22: OLS Regression of Village Level Antagonism Prevalence on Village Size, Population Density, Elevation, Wealth, Electricity, and Neighboring Village Animosity

	Dependent Variable: *Percent of Antagonistic Ties to All Social Ties*		
	Coef.	*S.E.*	*P*
Intercept	-12.475	8.324	0.136
Village Size	0.001	0.005	0.870
Population Density	-0.002	0.002	0.288
Elevation	0.040	0.018	0.029
$Elevation^2$	0.000	0.000	0.038
Wealth Index	0.674	0.827	0.416
Electricity	-1.097	1.176	0.353
Closest Village Animosity	**53.939**	**6.150**	**0.000**
Adjusted R^2		0.337	
N		176	

OLS regression of antagonism prevalence on village size, population density, elevation, wealth index, electricity, and animosity level of the geographically closest village (“as the crow flies”).

Supplementary Table S23: Visualization of the Heterogenous Triad Census (Census of Directed Triads with Heterogeneous Ties) and Total Observations by Triad Class Across 176 Villages

Visualization	Class Name	Total Observations (#)
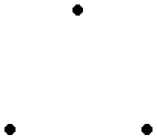	$B_3F_0E_0U_0S_0$	182,722,667
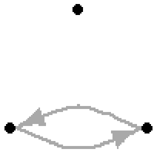	$B_2F_1E_0U_0S_1$	2,877,609
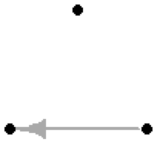	$B_2F_1E_0U_0S_0$	11,802,090
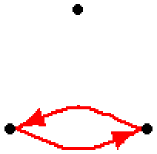	$B_2F_0E_1U_0S_1$	61,125
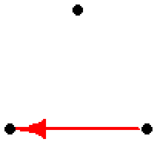	$B_2F_0E_1U_0S_0$	2,441,881
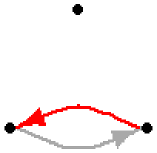	$B_2F_0E_0U_1S_0$	74,439
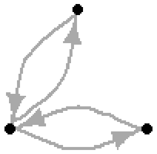	$B_1F_2E_0U_0S_5$	14,872

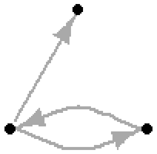	$B_1F_2E_0U_0S_4$	70,316
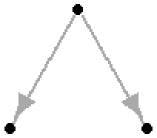	$B_1F_2E_0U_0S_3$	89,116
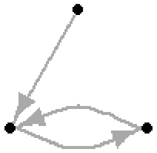	$B_1F_2E_0U_0S_2$	89,209
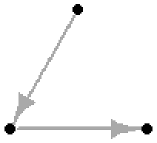	$B_1F_2E_0U_0S_1$	150,991
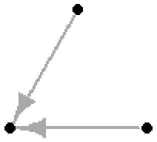	$B_1F_2E_0U_0S_0$	131,973
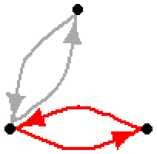	$B_1F_1E_1U_0S_8$	956
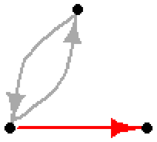	$B_1F_1E_1U_0S_7$	20,378
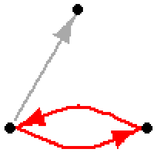	$B_1F_1E_1U_0S_6$	1,951
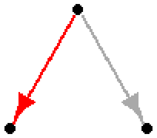	$B_1F_1E_1U_0S_5$	46,163

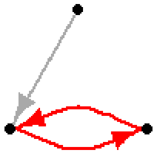	$B_1F_1E_1U_0S_4$	2,236
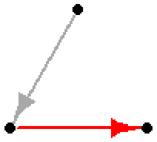	$B_1F_1E_1U_0S_3$	34,081
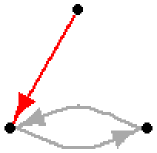	$B_1F_1E_1U_0S_2$	20,947
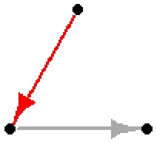	$B_1F_1E_1U_0S_1$	34,744
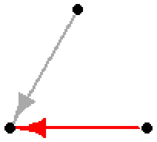	$B_1F_1E_1U_0S_0$	51,387
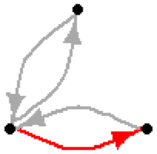	$B_1F_1E_0U_1S_5$	765
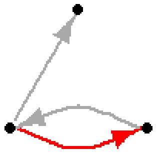	$B_1F_1E_0U_1S_4$	1,296
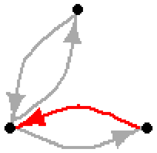	$B_1F_1E_0U_1S_3$	584
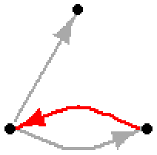	$B_1F_1E_0U_1S_2$	1,363

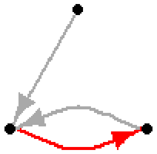	$B_1F_1E_0U_1S_1$	1,772
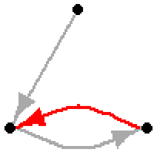	$B_1F_1E_0U_1S_0$	1,301
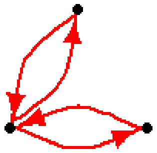	$B_1F_0E_2U_0S_5$	46
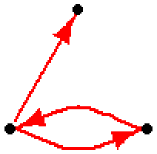	$B_1F_0E_2U_0S_4$	541
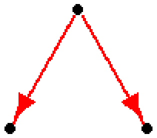	$B_1F_0E_2U_0S_3$	8,479
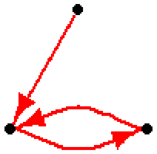	$B_1F_0E_2U_0S_2$	1,035
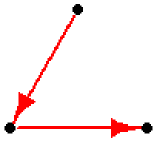	$B_1F_0E_2U_0S_1$	9,586
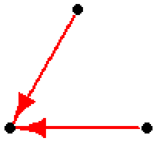	$B_1F_0E_2U_0S_0$	12,254
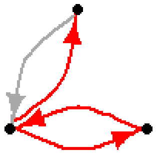	$B_1F_0E_1U_1S_5$	14

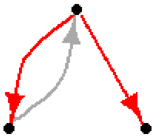	$B_1F_0E_1U_1S_4$	442
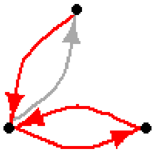	$B_1F_0E_1U_1S_3$	41
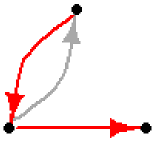	$B_1F_0E_1U_1S_2$	447
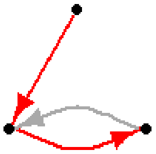	$B_1F_0E_1U_1S_1$	436
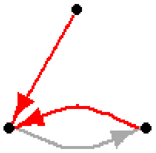	$B_1F\_0E_1U_1S_0$	564
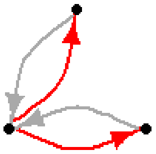	$B_1F_0E_0U_2S_2$	29
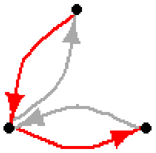	$B_1F_0E_0U_2S_1$	11
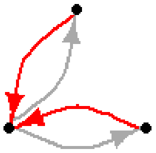	$B_1F_0E_0U_2S_0$	9
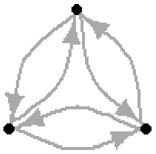	$B_0F_3E_0U_0S_6$	2,204

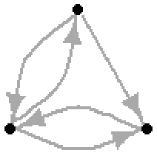	$B_0F_3E_0U_0S_5$	7,836
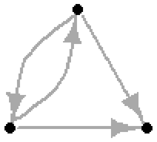	$B_0F_3E_0U_0S_4$	6,697
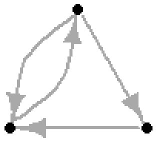	$B_0F_3E_0U_0S_3$	5,807
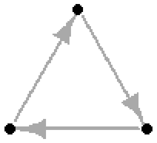	$B_0F_3E_0U_0S_2$	1,731
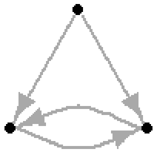	$B_0F_3E_0U_0S_1$	9,261
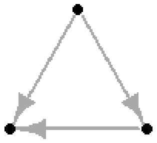	$B_0F_3E_0U_0S_0$	20,906
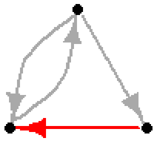	$B_0F_2E_1U_0S_{14}$	9
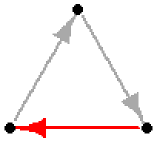	$B_0F_2E_1U_0S_{13}$	61
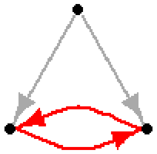	$B_0F_2E_1U_0S_{12}$	94

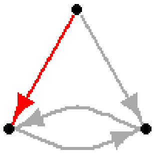	$B_0F_2E_1U_0S_{11}$	230
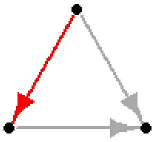	$B_0F_2E_1U_0S_{10}$	582
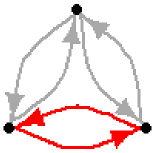	$B_0F_2E_1U_0S_9$	48
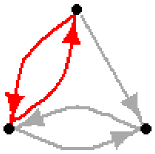	$B_0F_2E_1U_0S_8$	789
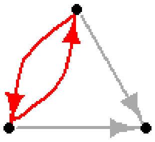	$B_0F_2E_1U_0S_7$	64
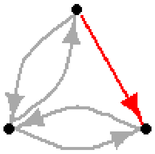	$B_0F_2E_1U_0S_6$	422
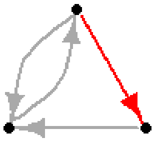	$B_0F_2E_1U_0S_5$	738
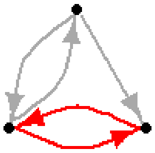	$B_0F_2E_1U_0S_4$	68
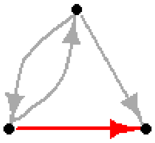	$B_0F_2E_1U_0S_3$	824

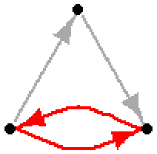	$B_0F_2E_1U_0S_2$	2,319
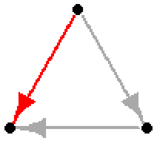	$B_0F_2E_1U_0S_1$	1,818
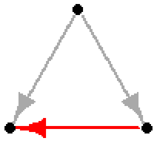	$B_0F_2E_1U_0S_0$	1,683
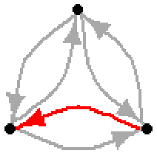	$B_0F_2E_0U_1S_8$	45
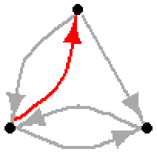	$B_0F_2E_0U_1S_7$	124
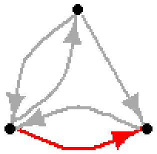	$B_0F_2E_0U_1S_6$	54
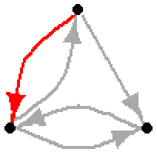	$B_0F_2E_0U_1S_5$	47
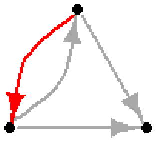	$B_0F_2E_0U_1S_4$	173
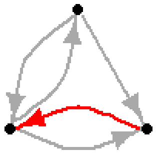	$B_0F_2E_0U_1S_3$	85

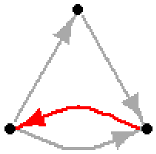	$B_0F_2E_0U_1S_2$	141
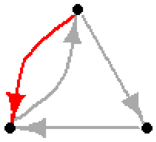	$B_0F_2E_0U_1S_1$	63
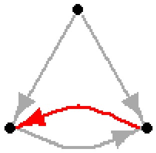	$B_0F_2E_0U_1S_0$	185
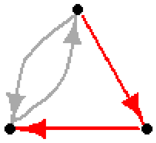	$B_0F_1E_2U_0S_{14}$	34
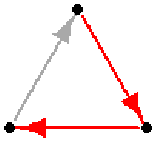	$B_0F_1E_2U_0S_{13}$	130
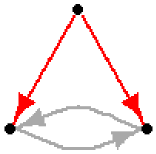	$B_0F_1E_2U_0S_{12}$	36
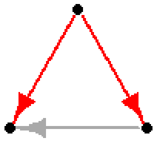	$B_0F_1E_2U_0S_{11}$	132
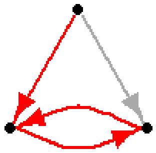	$B_0F_1E_2U_0S_{10}$	85
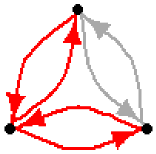	$B_0F_1E_2U_0S_9$	108

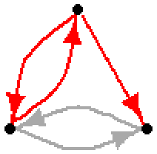	$B_0F_1E_2U_0S_8$	675
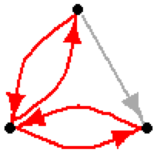	$B_0F_1E_2U_0S_7$	83
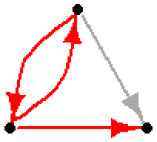	$B_0F_1E_2U_0S_6$	263
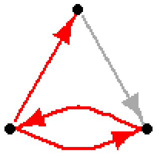	$B_0F_1E_2U_0S_5$	308
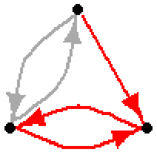	$B_0F_1E_2U_0S_4$	1,158
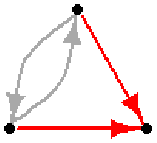	$B_0F_1E_2U_0S_3$	2,155
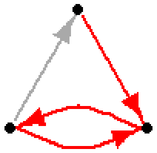	$B_0F_1E_2U_0S_2$	142
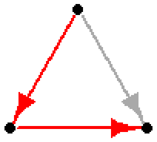	$B_0F_1E_2U_0S_1$	653
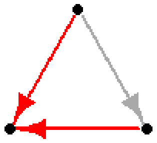	$B_0F_1E_2U_0S_0$	1,445

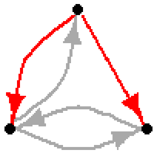	$B_0F_1E_1U_1S_{17}$	4
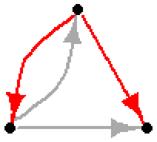	$B_0F_1E_1U_1S_{16}$	9
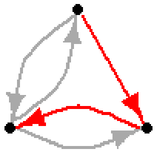	$B_0F_1E_1U_1S_{15}$	11
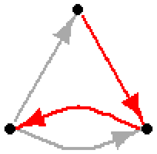	$B_0F_1E_1U_1S_{14}$	4
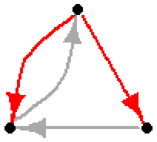	$B_0F_1E_1U_1S_{13}$	6
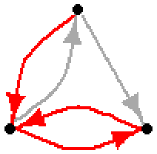	$B_0F_1E_1U_1S_{12}$	7
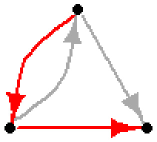	$B_0F_1E_1U_1S_{11}$	27
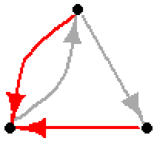	$B_0F_1E_1U_1S_{10}$	47
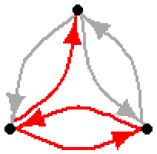	$B_0F_1E_1U_1S_9$	53

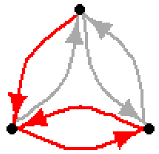	$B_0F_1E_1U_1S_8$	93
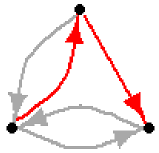	$B_0F_1E_1U_1S_7$	15
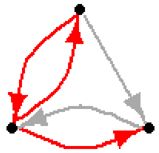	$B_0F_1E_1U_1S_6$	23
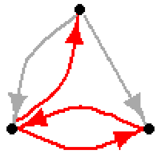	$B_0F_1E_1U_1S_5$	50
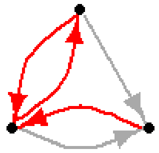	$B_0F_1E_1U_1S_4$	3
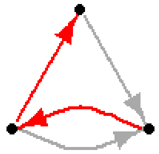	$B_0F_1E_1U_1S_3$	24
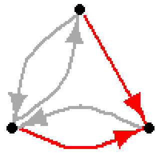	$B_0F_1E_1U_1S_2$	24
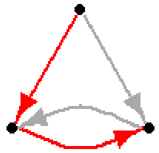	$B_0F_1E_1U_1S_1$	24
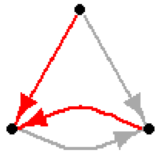	$B_0F_1E_1U_1S_0$	82

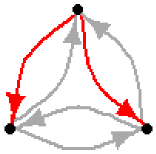	$B_0F_1E_0U_2S_6$	4
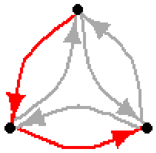	$B_0F_1E_0U_2S_5$	0
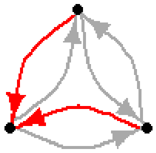	$B_0F_1E_0U_2S_4$	4
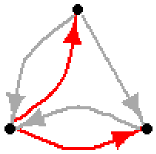	$B_0F_1E_0U_2S_3$	8
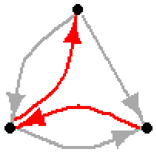	$B_0F_1E_0U_2S_2$	2
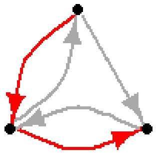	$B_0F_1E_0U_2S_1$	2
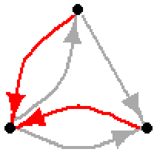	$B_0F_1E_0U_2S_0$	4
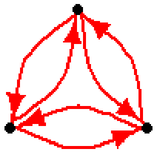	$B_0F_0E_3U_0S_6$	0
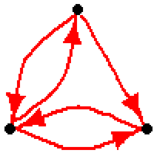	$B_0F_0E_3U_0S_5$	1

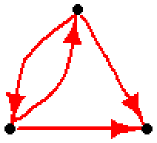	$B_0F_0E_3U_0S_4$	11
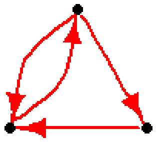	$B_0F_0E_3U_0S_3$	9
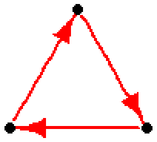	$B_0F_0E_3U_0S_2$	21
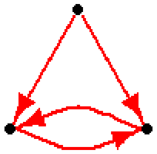	$B_0F_0E_3U_0S_1$	29
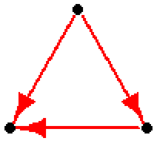	$B_0F_0E_3U_0S_0$	414
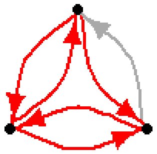	$B_0F_0E_2U_1S_8$	0
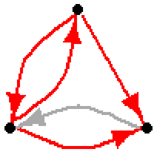	$B_0F_0E_2U_1S_7$	1
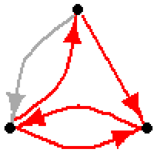	$B_0F_0E_2U_1S_6$	0
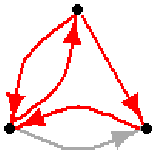	$B_0F_0E_2U_1S_5$	0

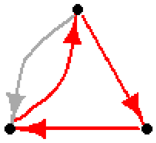	$B_0F_0E_2U_1S_4$	2
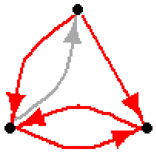	$B_0F_0E_2U_1S_3$	3
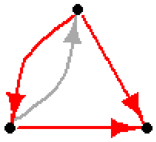	$B_0F_0E_2U_1S_2$	13
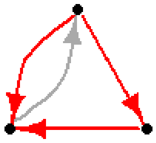	$B_0F_0E_2U_1S_1$	12
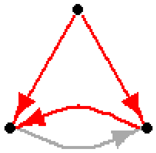	$B_0F_0E_2U_1S_0$	32
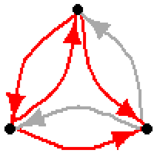	$B_0F_0E_1U_2S_6$	0
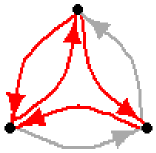	$B_0F_0E_1U_2S_5$	0
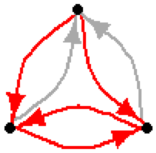	$B_0F_0E_1U_2S_4$	0
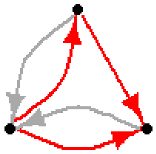	$B_0F_0E_1U_2S_3$	4

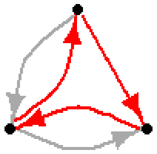	$B_0F_0E_1U_2S_2$	0
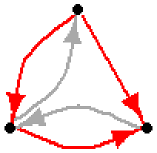	$B_0F_0E_1U_2S_1$	1
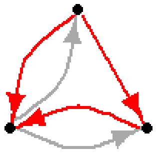	$B_0F_0E_1U_2S_0$	1
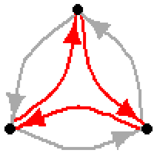	$B_0F_0E_0U_3S_1$	0
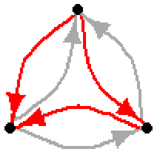	$B_0F_0E_0U_3S_0$	0

Complete census of 138 triad classes in a directed network with positive (gray) and negative (red) ties (column 1), with a consistent naming scheme in column 2 (see SI). We report the total number of observations of the triad type across villages in column 3.
